# Effect of Corrosive Medium and Surface Defect-Energy on Corrosion Behavior of Rolled ZK61M Alloy

**DOI:** 10.3390/ma15124091

**Published:** 2022-06-09

**Authors:** Jie Sun, Wenxiang Zhao, Pei Yan, Kaijie Chen, Li Jiao, Tianyang Qiu, Xibin Wang

**Affiliations:** 1 School of Mechanical Engineering, Beijing Institute of Technology, No. 5 Zhongguancun South Street, Haidian District, Beijing 100081, China; sj_bit@163.com (J.S.); bit_chenkaijie@163.com (K.C.); 2 Key Laboratory of Fundamental Science for Advanced Machining, Beijing Institute of Technology, No. 5 Zhongguancun South Street, Haidian District, Beijing 100081, China; zhaowx@bit.edu.cn (W.Z.); jiaoli@bit.edu.cn (L.J.); qiutianyangustu@126.com (T.Q.); cutting0@bit.edu.cn (X.W.)

**Keywords:** rolled ZK61M alloy, corrosion behavior, corrosive medium, surface defect-energy

## Abstract

Magnesium alloys have been widely used as lightweight engineering structural materials, but their service performances are severely restricted by corrosion failure. In this paper, the influence of corrosive medium and surface defect energy on the corrosion behavior of rolled ZK61M alloy was investigated. The corrosion tests were conducted in different concentrations of sodium chloride solution for different durations, and the polarization curves and electrochemical impedance spectroscopy were reported. The surface morphology of rolled ZK61M alloy before and after corrosion tests were analyzed. The results showed that the corrosion tendency became stronger with the increase of the concentration of corrosive medium and the number of surface defects of ZK61M alloy. Moreover, the initial corrosion pattern was the pitting caused by micro galvanic corrosion at the surface defect, which gradually developed into uniform corrosion. Furthermore, the main damage occurred at the grain boundary, resulting in the destruction of grain bonding force and the removal of material along the rheological layer. The oxidation corrosion mechanism was mainly the anodic dissolution mechanism.

## 1. Introduction

Due to a series of excellent properties such as low specific gravity, high specific strength and specific stiffness, good thermal conductivity and excellent machining performance, magnesium alloys are widely used as lightweight engineering structural materials in aerospace, automotive and other industries [1,2,3,4]. As a kind of Mg-Zn-Zr wrought magnesium alloy, ZK61M alloy has comprehensive properties and has been used as the key material for many structural components [5]. However, the industrial applications of ZK61M alloy are still restricted by its relatively poor corrosion resistance [6,7,8,9,10]. The corrosion process of magnesium can be regarded as a spontaneous process of electrochemical and chemical reactions. The surface of corroded magnesium is oxidized to porous magnesium oxide or magnesium hydroxide, which has poor corrosion resistance and cannot protect the matrix [11,12,13].

Many researchers have conducted investigations into the corrosion behavior of magnesium. Basically, the atmospheric corrosion of magnesium alloys is the most common form of damage [14]. Cui et al. [15] studied the corrosion behavior of AZ31 magnesium alloy exposed to a tropical marine atmosphere, and the results suggested that the corrosion rate changed periodically with the deposition rate of Cl^−^ and the corrosion developed from pitting to overall corrosion. Furthermore, magnesium alloys are easy to corrosion in most organic acids, inorganic acids and neutral media [16,17,18]. Cui et al. [19] investigated the corrosion properties of ZK61 magnesium alloy, which was immersed in simulated body fluid. The results showed that galvanic couples corrosion and dissolution of the protective film were the main reasons for poor corrosion resistance of ZK61 alloy. Pu et al. [20] conducted an immersion test of AZ31B magnesium alloy in 5 wt.% NaCl solution, which suggested that crystallographic orientations and grain size were the important factors determining the corrosion resistance. Song et al. [21] studied the corrosion behavior of pure Mg prepared by equal channel angular pressing (ECAP) in NaCl solution. The results showed that the strain-induced grain refinement with more crystalline defects weakened the corrosion resistance of pure Mg.

Magnesium and its alloys exhibit the negative difference effect, which plays an important role in the process of magnesium corrosion [14]. It refers to the increasing rate of hydrogen evolution from the magnesium surface under anodic polarization [22,23,24,25,26]. Based on the negative difference effect, many corrosion theories of magnesium alloys were established, which mainly included three types: (1) Low-valence Mg^+^ reaction theory [27]; (2) Dissolution and rupture theory of protective film [28]; (3) Second phase corrosion theory [29]. Abbott et al. [30] studied the corrosion behavior and corrosion resistance of magnesium alloys with Ce content. It was found that Ce content significantly affected the corrosion potential and corrosion current, and the increase in volume fraction of Mg_12_Ce would improve the corrosion rate. Although there is already singificant research in magnesium alloys corrosion, there is no unified corrosion theory due to the variety of materials and served environment.

These investigations have partially revealed the corrosion behavior of magnesium, such as corrosion, form in different mediums, methods to improve corrosion performance, etc. However, the interaction mechanism between corrosive medium and surface defect of magnesium alloys is less studied. This paper focused on the effect of corrosive medium and defect-energy on corrosion behavior and mechanisms of rolled ZK61M alloy. Corrosion tests in different concentrations of Cl^−^ solution with various duration were carried out and the corrosion behavior and mechanism were discussed. The experiment results and theoretical equations can be useful to gain a better understanding of the corrosion tendency of rolled ZK61M alloy.

## 2. Methodology of Research

### 2.1. Workpiece Material

**Figure 1:**
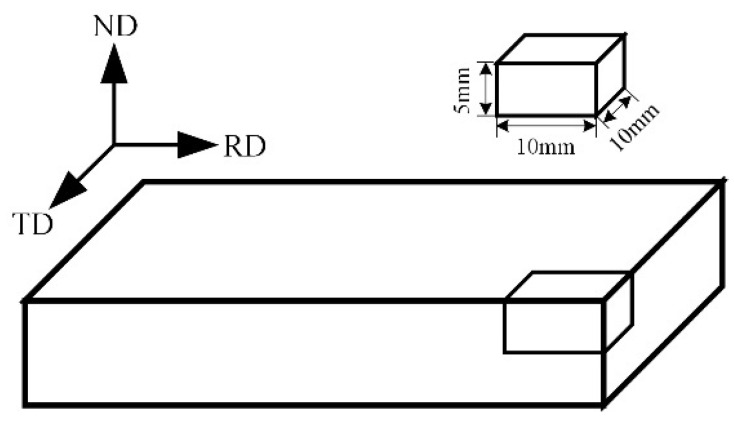
Schematic diagram of rolling plate sampling (ND: Normal Direction, TD: Transverse Direction, RD: Rolling Direction).

The workpiece used in this work was prepared from hot-rolled ZK61M alloy. Chemical compositions were shown in Table 1. To facilitate the operation and calculation of the electrochemical analysis, the samples were cut into cubes sized 10 mm × 10 mm × 5 mm, leaving an exposed area of 1 cm^2^. The surface perpendicular to the ND was used as the etched and observation surface as shown in Figure 1. In the electrochemical test, only the tested electrode surface was exposed to the corrosion solution, and the rest of the electrode was insulated with epoxy resin. Specimens were ground to 1000# using the SiC paper, cleaned with ethanol and dried by flowing cool air.

### 2.2. Corrosion Test

Corrosion behavior of ZK61M alloy was investigated by constant immersion tests in NaCl solution. The samples were suspended in the corrosion tank and the bottom was immersed in the solution. In order to study the influence of different concentrations and durations on corrosion behavior, immersion tests with different concentrations and durations were conducted. The prepared samples were immersed in different Cl^−^ concentrations of 0.2 mol/L, 0.4 mol/L, 0.6 mol/L, 0.8 mol/L and 1.0 mol/L, respectively. The corrosion duration was 4 h, 8 h, 18 h and 36 h, with the non-corrosive specimen as the control group. All the immersion tests were conducted at room temperature and repeated three times under each condition.

### 2.3. Electrochemical Analysis

Electrochemical workstation (Versa STAT MC, America) with three-electrode system was established to study the corrosion resistance of the immersed specimens. The saturated calomel was used as the reference electrode, the platinum plate acted as the counter electrode and the specimens with the tested areas of 1 cm^2^ served as the working electrode. Electrochemical tests mainly included the potentiodynamic polarization test and electrochemical impendence spectroscopy test (EIS), which were used to compare the corrosion resistance of samples. Moreover, the tests could be measured only after the open circuit potential (OCP) was stabilized. The scanning range of voltage was selected as −0.3~0.3 V, and the scanning rate of electrode potential was 0.33 mV/min. The scanning frequency range was 0.01 Hz~100 kHz, and the applied AC amplitude was 5 mV. All the tests were conducted at room temperature and carried out three times to ensure repeatability.

### 2.4. Microstructural Characterization

The original samples were analyzed from ND by metallographic microscope and scanning electron microscopy (SEM, QUANTAFEG 250, FEI Inc., Hillsboro, OR, USA). The phase structure and grain size distribution were analyzed by electron backscattered diffraction (EBSD), and the component energy spectrum was studied by energy dispersive spectroscopy (EDS). In order to study the surface morphology and corrosion mechanism, the surface and cross-section of the corroded alloy were analyzed by SEM.

## 3. Results

### 3.1. Microstructure

**Figure 2:**
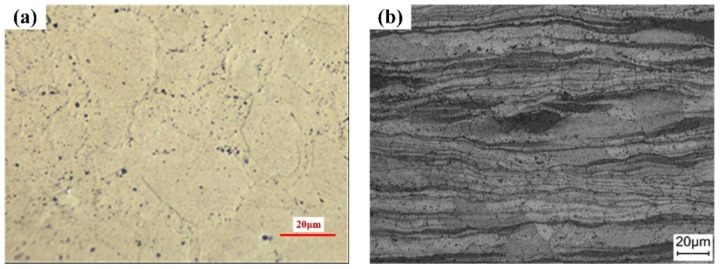
(**a**) Metallographic structure and (**b**) SEM of original ZK61M alloy from ND.

**Figure 3:**
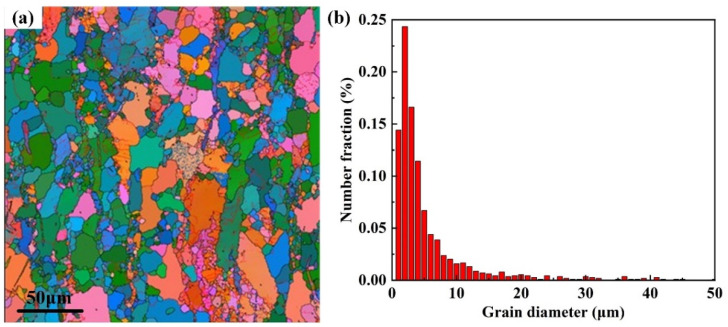
(**a**) EBSD Euler diagram of the original state of ZK61M alloy, and (**b**) Statistic of grain size in test section of ZK61M alloy.

The original microstructure of hot-rolled ZK61M alloy from ND is shown in Figure 2. The metallographic structure of the sample is a light-colored substrate, and there are black precipitates clustered at the sharp corners of grain boundaries. It can be seen from Figure 2b that the surface presents an irregular streamline, which is related to the rolling process. Figure 3 shows the Euler diagram and statistics of the grain size from the EBSD analysis. The grain boundaries are clearly visible. In addition to some coarse grains, a large number of smaller grains are distributed in the microstructure. Figure 3b shows that more than half of the grains are smaller than 5 μm in size and a small number of grains are over 15 μm in size. This indicates that the deformation is not uniform and there are a few unrefined grains in the rolling process. Moreover, the plastic deformation makes the grains refined and produces a large number of grain boundaries. The accumulation of dislocations at grain boundaries results in high dislocation energy. Corrosion is directly related to the increased prevalence of grain boundaries and dislocations in the microstructure [31].

**Figure 4:**
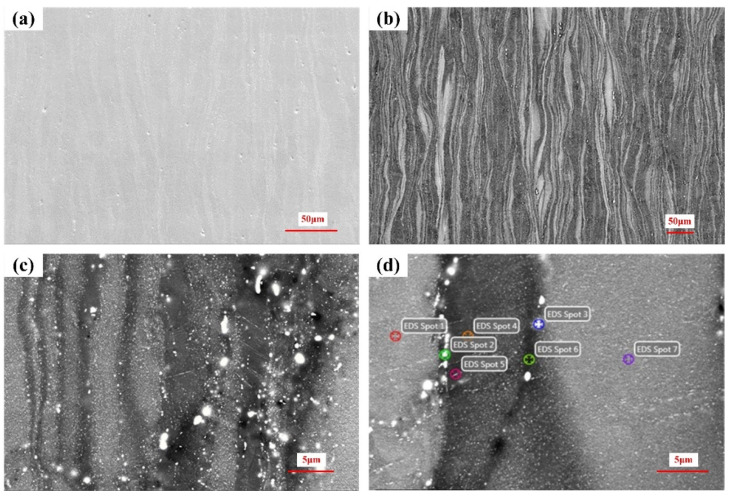
(**a**) low-multiple, (**b**) medium-multiple, (**c**) high-multiple SEM morphology and (**d**) EDS of original ZK61M alloy.

The samples are analyzed by SEM and EDS in the direction perpendicular to TD, as shown in Figure 4. It can be seen from Figure 4a,b that there are some needle-like pores of different sizes, presenting lamellar microstructure. Figure 4c,d shows the morphology of the rheological layer at high magnification. The white point-like precipitates can be found in the microstructure. Seven points are selected and analyzed by EDS, and the chemical element content shows in Table 2. It can be concluded that the white spots are precipitates rich in Zn and Zr. Generally, the second phase is an important factor affecting the corrosion resistance of magnesium alloys. The potential difference between the second phase and the substrate causes galvanic corrosion. With the increase in potential difference, the corrosion becomes more intense [32].

### 3.2. Polarization Curves

**Figure 5:**
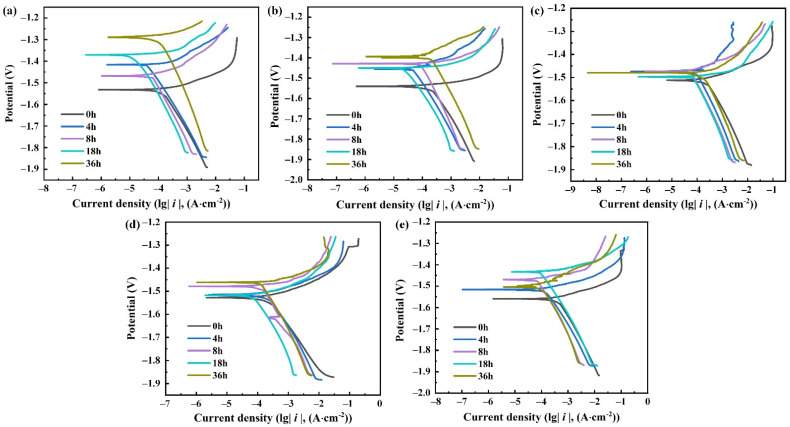
Polarization curves of ZK61M alloy in Cl^−^ solutions with different concentrations: (**a**) 0.2 mol/L, (**b**) 0.4 mol/L, (**c**) 0.6 mol/L, (**d**) 0.8 mol/L and (**e**) 1.0 mol/L.

Figure 5 depicts the cathodic and anodic polarization curves corroded in Cl^−^ solution for 4 h, 8 h, 18 h and 36 h, with the non-corrosive specimen as the control group. With the prolongation of corrosion time, the corrosion potential of the corroded electrode gradually increases. Corrosion is most likely to occur at the beginning of the experiment because the exposed surface is not covered by oxide film yet. With the progress of corrosion, the oxidation films and corrosion products are generated on the surface, resulting in the improvement of corrosion resistance. The corrosion is hindered, and the corrosion rate is reduced [33].

It can be found from the curves that there are strong polarization regions in cathode polarization curves, while there are none in the anode polarization curves. Moreover, the anode and cathode branches are not symmetrical in the process of reaction. The increasing rate of the current density of the cathode branch curves with the negative shift of corrosion potential is less than that of the corresponding anode curves with the positive shift. This is due to the negative difference effect of magnesium alloy and the corrosion of the maximum cathode and small anode [34,35].

**Figure 6:**
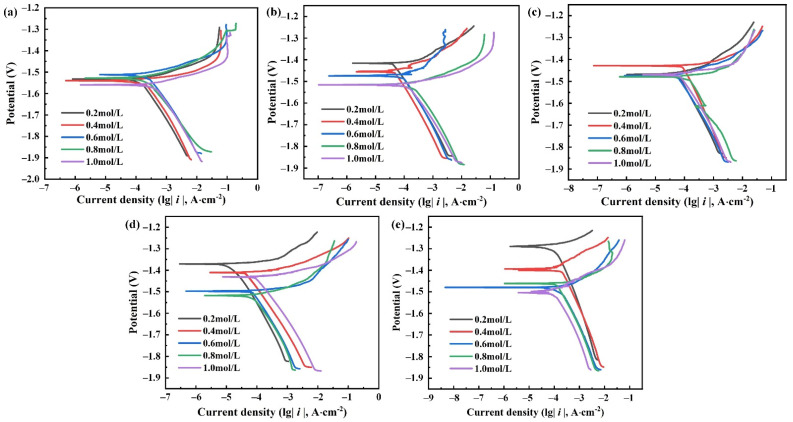
Polarization curves of ZK61M alloy with different time immersed in Cl^−^ solutions: (**a**) 0 h, (**b**) 4 h, (**c**) 8 h, (**d**) 18 h and (**e**) 36 h.

Figure 6 shows the polarization curves measured at the same time in 0.2 mol/L, 0.4 mol/L, 0.6 mol/L, 0.8 mol/L and 1.0 mol/L Cl^−^ solutions, respectively. There is a strong correlation between the corrosion potential and the concentration of corrosion solution. When the concentration of the corrosion solution increases, the corrosion potential of the electrode decreases. Similarly, there is a strong linear polarization region in the cathodic polarization curves, but it is not obvious in the anode polarization curves.

**Figure 7:**
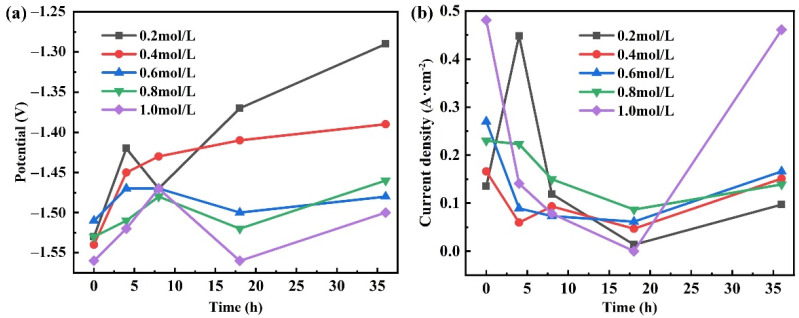
The change of the corrosion potential (**a**) and corrosion current density (**b**) in different solutions concentrations and duration of immersion.

Figure 7 shows the variation of the corrosion potential and corrosion current density with the extension of time in different concentrations of solutions. Figure 7a shows the variation of corrosion potential with duration. It can be found that the initial etching potential is related to the concentration of the solution. When the concentration of the solution is 0.2 mol/L and 0.4 mol/L, the corrosion potential of the corroded electrode has little difference. However, with the increase in the concentration of the solution, it is obvious that the corrosion potential of the corroded electrode decreases rapidly. Except for the concentration of 0.4 mol/L, the corrosion potential at other concentrations shows a similar trend with time. With the prolongation of time, the corrosion potential shows a trend of increase-reduce-increase. This is because at the initial stage of corrosion, the corrosion resistance is low due to the interface formed by the magnesium alloy and the solution is close to the ideal interface. Although with the extension of time, the oxide film forming on the surface of magnesium alloy gradually thickens, reducing the interface corrosion potential and increasing the corrosion resistance. This indicates that the original microstructure and surface state of ZK61M alloy have been completely destroyed [36]. Figure 7b shows the change of corrosion current with time. Similar to etching potential, the etching current density is the largest and the reaction rate is the fastest at the beginning.

### 3.3. Electrochemical Impedance Spectroscopy

**Figure 8:**
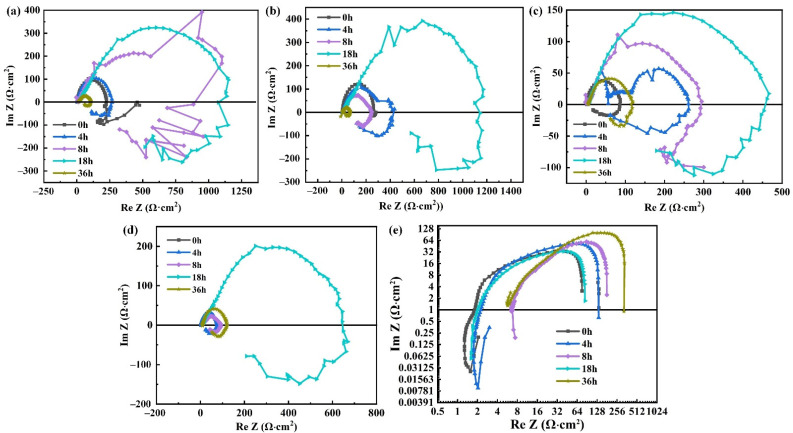
Electrochemical impedance spectroscopy of ZK61M alloy in different Cl^−^ concentrations: (**a**) 0.2 mol/L, (**b**) 0.4 mol/L, (**c**) 0.6 mol/L, (**d**) 0.8 mol/L and (**e**) 1.0 mol/L.

Figure 8 shows the electrochemical impedance spectroscopy of specimens in different Cl^−^ concentrations. It can be seen from Figure 8a–d that the curves under all conditions are composed of a capacitive and an inductive reactance arc. The diameters of curves also change regularly with the prolongation of corrosion time. When the surface of the magnesium alloy is not corroded, the diameter of the entire impedance spectroscopy is the smallest, indicating that the resistance of the corroded electrode system is the lowest [37]. With the increase of corrosion time, the diameter of the curve gradually increases, and the maximum diameter appears at the corrosion time of 18 h.

It can be found from Figure 8a,d that in the same corrosion time, the diameters of impedance spectrum obtained in a higher Cl^−^ concentration solution is much less than that in a lower concentration solution. In addition, as the concentration of Cl^−^ increases to 1.0 mol/L, the corrosion rate becomes very high. Therefore, the data is plotted in the logarithmic coordinates and the trend of the curves can be seen clearly.

### 3.4. Surface of the Corroded Alloys

**Figure 9:**
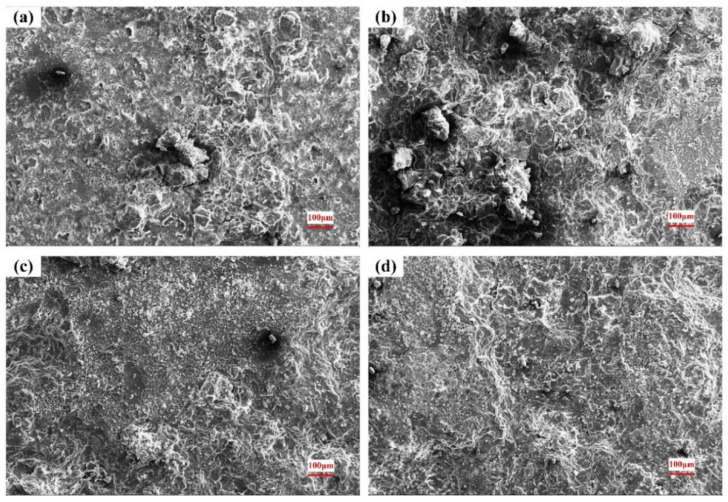
Surface morphology after corrosion in 0.2 mol/L Cl^−^ solution: (**a**) 4 h; (**b**) 8 h; (**c**) 18 h; (**d**) 36 h.

**Figure 10:**
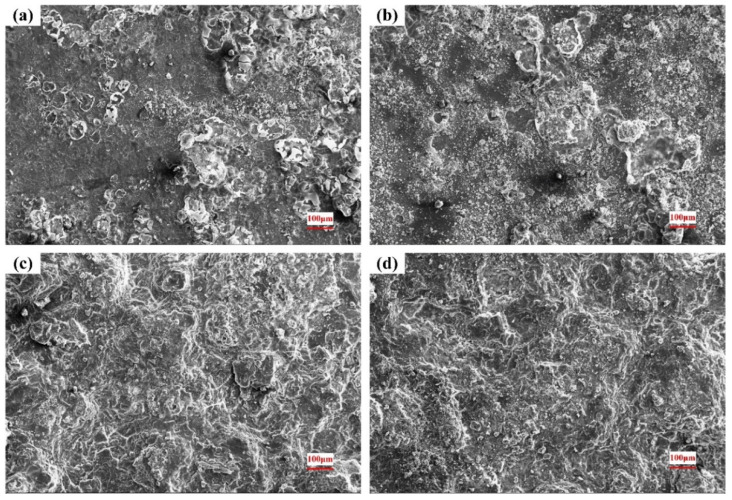
Surface morphology after corrosion in 0.6 mol/L Cl^−^ solution: (**a**) 4 h; (**b**) 8 h; (**c**) 18 h; (**d**) 36 h.

**Figure 11:**
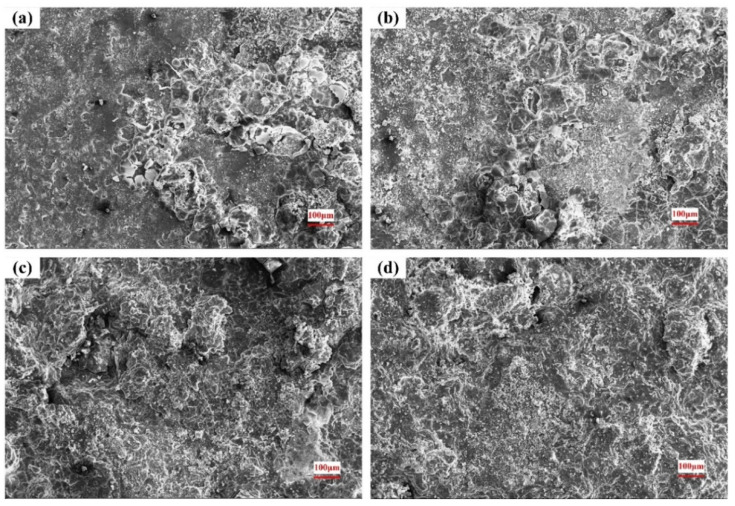
Surface morphology after corrosion in 1.0 mol/L Cl^−^ solution: (**a**) 4 h; (**b**) 8 h; (**c**) 18 h; (**d**) 36 h.

The corroded surface morphologies in 0.2 mol/L, 0.6 mol/L and 1.0 mol/L Cl^−^ solutions are shown in Figure 9, Figure 10 and Figure 11. As seen in Figure 9a, obvious dark etching spots and spherical bulges appear on the surface after a short period of corrosion. After 8 h, many corrosive products have accumulated in the original pitting pits. It can be seen from the morphology after 18 h that the corrosion products accumulated in the pitting gradually fall off, and the pitting becomes deeper and larger. Eventually, most of the substrate is shed, forming a large erosion pit.

Under the different Cl^−^ concentrations, the corrosion morphologies are similar, and the pitting corrosion gradually expands to uniform corrosion of the whole plane. When the concentration of the solution increases, the corrosion rate is accelerated and the corrosion degree is aggravated, as shown in Figure 10 and Figure 11.

Combined with the original microstructure and corroded surface morphology, it can be found that the corrosion starts to nucleate from the second phase on the substrate surface. As a strengthening phase, the second phase contains a large amount of Zr element, with high hardness and good corrosion resistance. Under the action of electrolyte solution, a micro-galvanic cell is formed in the phase boundary, which is easily dissolved by pitting corrosion. As the corrosion continues, the material around the pits continues to dissolve and the pits expand in all directions. As the corrosion products of magnesium alloy are relatively loose, local materials will fall off with the expansion of the corrosion pit, and the entire surface will be destroyed [38].

### 3.5. Cross-Section of the Corroded Alloys

**Figure 12:**
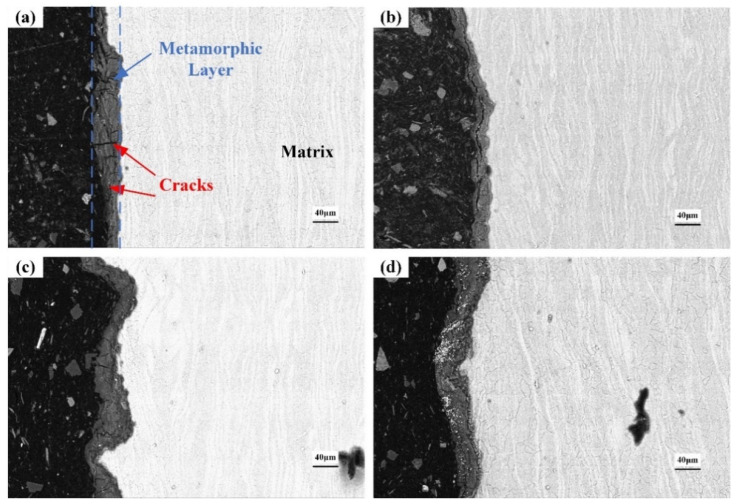
Cross-section morphology after corrosion in 0.2 mol/L Cl^−^ solution: (**a**) 4 h; (**b**) 8 h; (**c**) 18 h; (**d**) 36 h.

**Figure 13:**
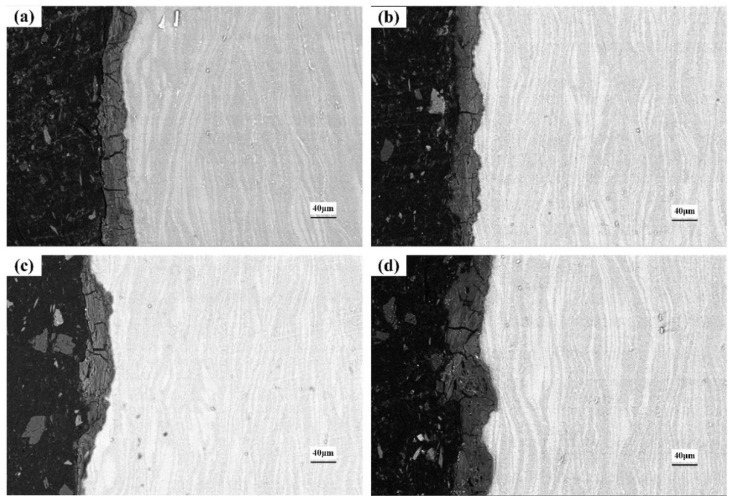
Cross-section morphology after corrosion in 0.6 mol/L Cl^−^ solution: (**a**) 4 h; (**b**) 8 h; (**c**) 18 h; (**d**) 36 h.

**Figure 14:**
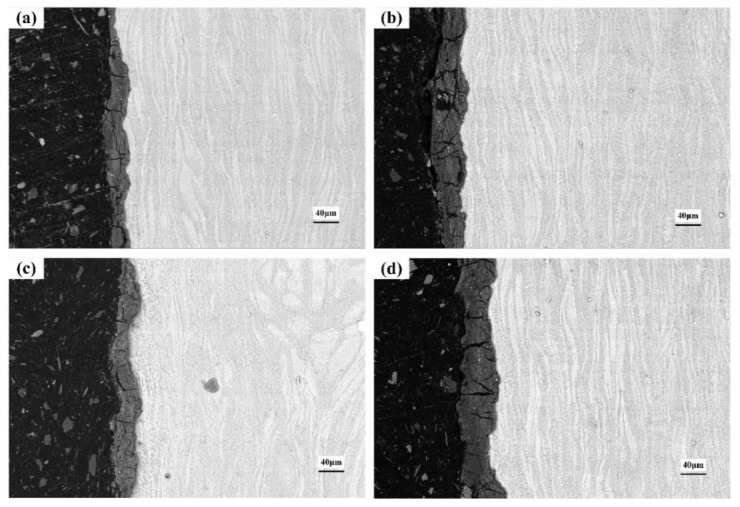
Cross-section morphology after corrosion in 1.0 mol/L Cl^−^ solution: (**a**) 4 h; (**b**) 8 h; (**c**) 18 h; (**d**) 36 h.

**Figure 15:**
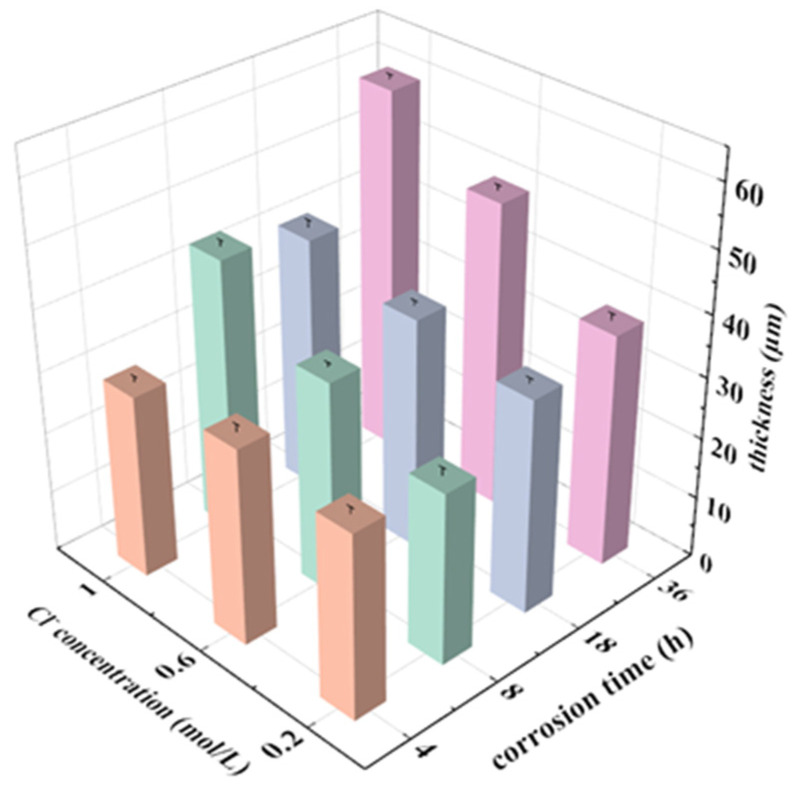
Thickness of the corrosion metamorphic layer in the different immersion tests.

Figure 12, Figure 13 and Figure 14 show the cross-section morphologies after different durations of corrosion in 0.2 mol/L, 0.6 mol/L and 1.0 mol/L Cl^−^ solution, respectively. The thickness of the corrosion metamorphic layer in the different immersion tests is shown in Figure 15. It can be seen that the thickness of the corrosion metamorphic layer gradually thickens with the increased Cl^−^ concentration and the extension of time. In addition, the distribution profile of these metamorphic layers completely coincides with the rolling rheological track of the surface. It shows that the corrosion is peeled from the surface to the inside layer by layer along the rheological track. There are short cracks along the grain boundary and perpendicular to the surface of the matrix at the interface between the corrosion metamorphic layer and the matrix.

## 4. Discussion

### 4.1. Corrosion Tendency

Under the action of electrostatic force and surface energy, the space charge layer is generated at the interface of two phases, forming the double electric layer structure. The structure and charge distribution of the two-phase interface have great influence on the electrochemical corrosion process.

**Figure 16:**
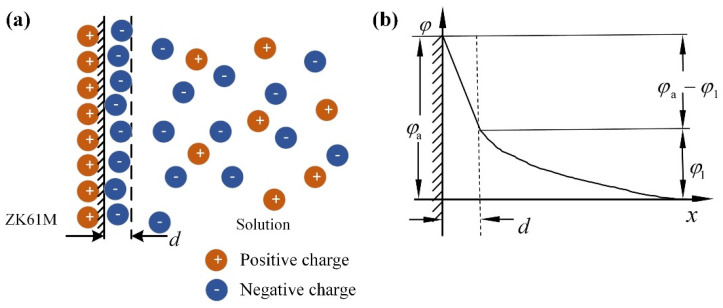
(**a**) Schematic diagram of ideal interface structure of ZK61M alloy/NaCl solution, (**b**) Schematic diagram of potential distribution as a function of interface distance.

A double-layer electrostatic model called GCS is established. ZK61M alloy with a smooth surface is placed in Cl^−^ solution, and the basic structure of its ideal interface structure can be obtained, as shown in Figure 16a. There are a lot of free electrons in the magnesium alloy. When a small amount of residual charge is concentrated at the interface, the distribution of free electrons is not disturbed. The remaining charges in the magnesium alloy are tightly arranged, and the potential at each point in the alloy is equal. Figure 16b is a schematic diagram of the potential distribution of this model as a function of interface distance.

Based on the interface structure model, the mathematical expressions of charge quantity and potential can be derived. It is assumed that there is only electrostatic force between solution ions and alloy surface. The thickness of the interfacial double layer is much less than the curvature radius of the alloy surface. The potential distribution of the double layer can be considered as a one-dimensional function in the X direction. According to Maxwell–Boltzmann distribution, it can be obtained that the ion concentration at the liquid layer at X from the magnesium alloy surface is:

(1) $$c_{+}=c\cdot \exp(-\frac{\varphi m}{RT})$$

(2) $$c_{-}=c\cdot \exp(\frac{\varphi m}{RT})$$

where $c_{+}$, $c_{-}$ are the concentrations of positive and negative ions at the liquid layer with potential of φ at distance of X from the surface of the magnesium alloy; c is the concentration of the solute; *R* is the Boltzmann constant (1.381 × 10^−23^ m^2^·kg·K^−1^·s^−2^); *T* is the absolute temperature and *m* is the number of charges on the particle.

In the liquid layer of X from the surface of the magnesium alloy, the volume charge density of the remaining charge is:

(3) $$\rho =mc_{+}-mc_{-}$$

(4) $$\rho =c\cdot m\left[\exp(-\frac{\varphi m}{RT})-\exp(\frac{\varphi m}{RT})\right]$$

where ρ is the volume charge density, *m* is the number of charges on the particles.

Poisson equation, Taylor formula and Gaussian integral are applied to integrate and simplify the above formulas. The mathematical expression of the GCS double electric layer model for the ideal interface of ZK61M alloy in Cl^−^ solution is obtained as follow:

(5) $$q=\sqrt{8cRT\varepsilon_{0}\varepsilon_{r}}\sinh\left(\frac{\varphi_{1}m}{2RT}\right)$$

Under the effect of the potential difference between the two sides of the interface, the magnesium alloy atoms with higher atomic kinetic energy will overcome the lattice constraint. It can be seen from the equation that the potential difference of the dispersion layer is related to the surface charge density of the alloy and the concentration of the solution.

In the previous discussion, there are many stacking errors, segregation, texture and lattice distortion on the surface layer of magnesium alloy, as well as the existence of other adsorbed substances. Therefore, it is necessary to analyze the influence of surface defects on corrosion. Generally, the corrosion tendency of metal is judged by the thermodynamic energy difference. The potential of the corrosion system can be used to describe the corrosion tendency.

The energy *E_e_*_1_ of unit length mixed dislocation is:

(6) $$E_{e1}=\frac{Gb^{2}}{4\pi }\left(\cos^{2}\varphi +\frac{\sin^{2}\varphi }{1-\nu }\right)\ln\frac{2R}{b}$$

The Nernst formula of electrode reaction under ideal conditions is as follows:

(7) $$E_{a}=E_{{}^{0}}+\frac{\mathrm{RT}}{nF}\ln a$$

where a is the activity of the metal electrode, $E_{a}$ is electrochemical potential, $E_{{}^{0}}$ is the standard electrochemical potential of a metal, F is Faraday constant, n is the valence number of metal ions in the electrode reaction, *R* is the gas constant and *T* is the absolute temperature.

When magnesium alloy with the number of surface dislocation defects of *N* is in the solution of ion concentration of *c*, it can be assumed that the electrochemical potential is *E_C_*, which satisfies the following equation:

(8) $$E_{c}=E_{{}^{0}}+\frac{RT}{nF}\ln a+E_{sd}+E_{wc}$$

where $E_{c}$ is electrochemical potential under actual conditions, $E_{sd}$ is the electrochemical potential of a double layer in a solution of concentration *c*, $E_{wc}$ is electrochemical potential considering material surface dislocations.

Based on the Nernst formula, the interface double electric layer model and the dislocation energy are introduced, and the electrochemical potential of the corrosion system under the influence of a surface fault is deduced as follows:

(9) $$E_{c}=E_{{}^{0}}+\frac{RT}{nF}\ln a+\varphi_{1}+N\cdot E_{e1}$$

(10) $$E_{c}=E_{{}^{0}}+\frac{RT}{nF}\ln a+\frac{2RT}{F}\ln\sqrt{\frac{2q^{2}+8cRT\varepsilon_{0}\varepsilon_{r}}{8cRT\varepsilon_{0}\varepsilon_{r}}}+N\frac{Gb^{2}}{4\pi }\left(\cos^{2}\varphi +\frac{\sin^{2}\varphi }{1-\nu }\right)\ln\frac{2R}{b}$$

According to Equation (10), the electrochemical potential of the corrosion system continuously increases with the increase in the concentration of the corrosion solution and the number of defects on the material surface. As the corrosion potential of the corrosion reaction system becomes lower and lower, the corrosion tendency becomes stronger and stronger.

### 4.2. Corrosion Behavior and Mechanism

**Figure 17:**
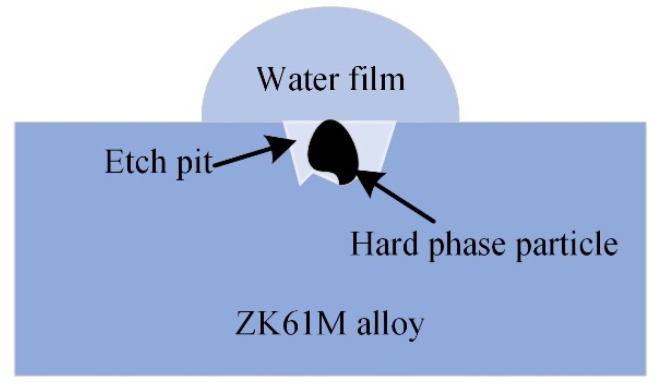
Schematic diagram of initial pitting corrosion mechanism.

According to the results of corrosion tests and the theory of the double electric layer model, the corrosion behavior and mechanism of hot-rolled ZK61M alloy can be summarized as the following stages:

The first stage is rapid corrosion. The material is in direct contact with the etching solution and the solid-liquid interface rapidly forms a double-layer structure. The surface has a very high electrochemical potential due to the high surface energy and the potential difference generated by the double-layer electric field. The energy and double-layer potential difference in the defect area such as the second phase particles are significantly different from those in other areas. A small electrochemical corrosion galvanic cell system will be formed around these defects, resulting in pitting. The rapid pitting corrosion causes the corrosion area to expand, as shown in Figure 17. H. Kalb et al. [39] studied the corrosion of magnesium alloys during exposure to simulated body fluid, indicating that the microgalvanic processes dominate degradation morphology and formation of the corrosion/conversion layer. After that, the corrosion changes from pitting to uniform corrosion, and the thickness of the corrosion oxide layer gradually increases. The corrosion metamorphic layer can hinder the material transfer at the corrosion electrode, thus reducing the corrosion speed and producing a certain protective effect on the substrate. As reported by Man et al. [40], the corrosion product layer protected the sample from corrosion, but this protective ability weakened due to the cracks.

The corrosion rate of the second stage is lower than that of the first stage. Due to the corrosion reaction, the entire surface of the material has been covered by an oxide layer. In addition to the deep pitting pits in the local part of the material due to uneven energy structure, the other areas are uniformly corroded. The corrosion metamorphic layer is also becoming thicker. The outer corrosive layer is too loose and easily falls off, while the inner layer is still tight, but also accompanied by a large number of micro-cracks.

**Figure 18:**
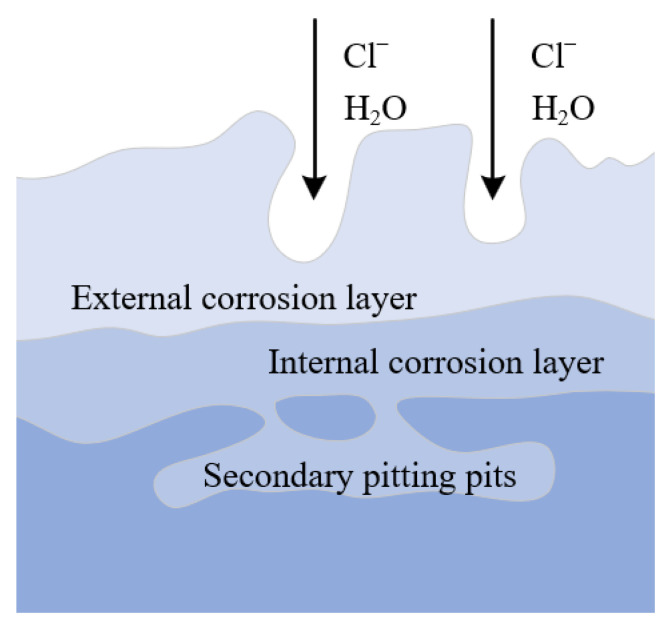
Schematic diagram of mechanism of new pitting induced by late corrosion.

The third stage is stable corrosion. After the first and second corrosion stages, the loose oxide film has fallen off. The entire surface is covered with a thick layer of corrosion. However, due to the loose metamorphic layer, Cl^−^ can penetrate the oxide film and react with the matrix to induce new pitting locally. Moreover, due to the difference of local solution concentration, pitting corrosion gradually develops into pore corrosion. The corrosion mechanism is shown in Figure 18.

## 5. Conclusions

In this paper, the corrosion behavior and mechanism of rolled ZK61M alloy have been investigated, and the main conclusions can be addressed as follows:

(1) With the increase of the concentration of corrosive medium and the number of material surface defects, the corrosion tendency becomes stronger.
(2) The corrosion current density decreases and tends to be stable with the increase of duration due to the obstruction of the surface oxide film.
(3) Corrosion expands along grain boundaries and corrosion products fall off layer by layer along the rolled rheological layer.
(4) The corrosion is initially pitting caused by micro galvanic corrosion at the surface defect, and gradually changes into uniform corrosion. The oxidation corrosion mechanism is the anodic dissolution.

## Figures and Tables

**Table 1:** Chemical compositions of the experimental alloys (wt. %).

Zn	Zr	Mn	Impurity	Mg
5~6	0.8~1	0.05	<0.01	Bal.

**Table 2:** Chemical element content at each representative point (wt. %).

	Number	1	2	3	4	5	6	7
Element								
Mg		92.01	88.07	91.81	94.83	94.97	95.61	93.32
Zn		7.31	10.69	8.09	5.06	4.96	4.32	7.17
Zr		0.67	1.24	0.09	0.11	0.07	0.06	0.51

## Data Availability

All the data is available within the manuscript.
